# Family Support and Loneliness among Older Persons in Multiethnic Malaysia

**DOI:** 10.1155/2014/654382

**Published:** 2014-10-14

**Authors:** Jane Kimm Lii Teh, Nai Peng Tey, Sor Tho Ng

**Affiliations:** Population Studies Unit, Faculty of Economics and Administration, University of Malaya, 50603 Kuala Lumpur, Malaysia

## Abstract

This study investigates factors affecting older persons' state of loneliness in multiethnic Malaysia using data from the 2004 Malaysian Population and Family Survey, the first nationally representative sample in Malaysia. The study sample was extracted to include Malays, Chinese, Indians and other Indigenous groups aged 60 and above, and who had children (*n* = 1791). Cross tabulations and ordinal logistic regression methods were used in the analysis. Among the ethnic groups, older Malays were more likely than their Chinese and Indian counterparts to experience loneliness. Loneliness was found to be associated with age, marital status, education level, sources of income, health status, and physical limitations. Among older people, feelings of loneliness were inversely related with coresidence with adult children and participation in religious activities. Sociodemographic changes have eroded the traditional family support system for the elderly, while social security remains inadequate. This study shows the important role of family in alleviating loneliness among older people. Hence the need to promote and facilitate coresidence, as well as participation in religious activities, and a healthy lifestyle as a priority strategy is in line with the objectives of the National Policy for the Older People.

## 1. Introduction

Loneliness is prevalent among older persons [1–3]. Loneliness is a state of mind as “people can live rather solitary lives and not feel lonely, or they can have many social relationships and still feel lonely” [4]. Loneliness has also been described as the distress due to the inconsistency between ideal and perceived relationships [5], which, in turn has a great impact on health and the quality of life [6]. Persistent loneliness and being very lonely are detrimental to the well-being of an individual. Loneliness is found to be a precursor to psychological disorders, mental health problems, depression, and even suicide [2, 3, 5, 7–12].

Studies found that sociodemographic factors such as age and marital status influence loneliness [7, 13, 14]. Older persons with low educational attainment and income and the unemployed are likely to feel lonely as compared to those with higher education and income and who are working [4, 13, 15, 16]. Loneliness is also strongly associated with poor health. Loneliness increases with reduced cognitive function [14], reduced social activities, and higher physical limitations [15, 17]. Older persons with chronic stress [4], chronic diseases [18], and visual impairments [19] are also more likely to feel lonely as compared to those who do not have these conditions.

Social networks are of great importance in determining the quality of life and well-being of older persons [15, 20]. Apart from their spouse, adult children provide the most important support and social contact in old age. Adult children's more frequent contact, care and affection may lessen the feelings of loneliness among older persons [21–23]. Friends and neighbours may also provide emotional support and assistance in tasks such as transportation and the running of errands [24]. Older persons who are close with family members and have many friends are psychologically well-adjusted than those without these networks [25]. Nevertheless, higher levels of support may not always have positive outcomes [20]. An earlier study found that the prevalence of loneliness is more common in areas where living alone is rare and where there is strong integration within a community [26].

This study examines factors affecting loneliness, with emphasis on the influence of family and community engagement in ameliorating the feelings of loneliness among older persons in multiethnic and multicultural Malaysia. Specifically, this study aims to examine the correlates of loneliness in terms of (i) sociodemographic and socioeconomic characteristics, (ii) health and physical condition, (iii) various forms of support from adult children, and (iv) community engagement through religious and leisure activities.

This study relies on the convoy model of social relations to examine the influence of formal and informal support networks on an older person's emotional state, in particular the feelings of loneliness. This model describes how social support systems function throughout the life course and provides a framework of how people manage and maintain personal social network structures through life [27]. These structures also change in response to different situations as people pass through their life cycles [28]. Others have further added that social networks of older persons are conditioned by the social and cultural norms engrained in the societies they belong to [29].

Malaysia is a South-east Asian nation and constitutes of two regions (Peninsular Malaysia and East Malaysia) separated by the South China Sea. The Malays, Chinese, and Indians are the main ethnic groups in Peninsular Malaysia, whereas Indigenous people are concentrated in East Malaysia. Malaysian citizens consist of around 55% Malays, 24% Chinese, 7% Indians, 13% Indigenous people, and 1% of other ethnicity. These ethnic groups have diverse backgrounds, cultures, and religion. Islam is the national religion and around 61% of the Malaysian populations are Muslims [30]. All Malays are Muslims, around 76% of the Chinese are Buddhists and 9% are Christians, around 85% of the Indians are Hindus, and 8% are Christians. About half of the Indigenous people are Christians and 36% are Muslims [30].

The Government has always maintained that it is the duty of children to provide care and support for their aging parents [31, 32]. Traditionally, it has been the norm and cultural practice of all ethnic groups for children to repay their parents (*balas jasa*) [33]. However, Malaysia has undergone rapid demographic transition, with continuing decline in fertility and increasing life expectancy over several decades. Since independence from British rule in 1957, the total fertility rate has declined from 6.1 per woman to 2.1 in 2010 [34, 35]. The fertility rate among the Malays and other Indigenous groups remains considerably above replacement level (at around 2.8 children per woman) while that of the Chinese and Indians has gone below replacement level since 2010. During this period, life expectancy for males has increased from 55.8 years to 71.9 years, while that of the females has increased from 58.2 years to 76.6 years. The increasing number of older persons with diminishing family size will put more stress on traditional family support systems.

Financial support is a common form of support for older parents. Previous findings in Malaysia revealed that older Malaysians, especially those living in rural areas, largely depend on financial support from their children [36, 37]. Coresidence between older parents and adult children is a traditional form of support, as practised in many parts of Asia [38, 39]. This living arrangement ensures companionship and financial and emotional support for older parents, besides sharing the cost of living and domestic chores [38, 40].

## 2. Method

### 2.1. Data and Sample

Data for this study are taken from the nationally representative Malaysian Population and Family Survey (MPFS-4), conducted in 2004 by the National Population and Family Development Board (LPPKN) of Malaysia, with technical assistance from the Department of Statistics Malaysia. The MPFS-4 sample was selected using a stratified multistage sampling design and the main survey was fielded between July 2004 and September 2004. The Kish method was used in the selection of senior citizens (aged 50 and above) within the selected household. The Malaysian Population and Family Survey had been conducted every 10 years since 1974. MPFS-4, the latest in the series, was the first which covered Peninsular Malaysia as well as Sabah and Sarawak in East Malaysia. The survey had an overall response rate of about 80% of the senior sample [41–43].

We merged the three separate senior samples (Peninsular Malaysia, Sabah, and Sarawak) into one main data file. Then, we filtered to include only Malaysian citizens aged 60 and above and those who had children (96%). The few non-Malaysian and those with no children were excluded from the analysis because our main objective was to examine the role of family support on loneliness among older Malaysians.

### 2.2. Measures

Loneliness, the dependent variable is an ordered variable (*1 = not lonely, 2 = sometimes lonely, *and* 3 = always lonely*). Respondents were asked if they have ever felt lonely. Respondents who gave an affirmative response were subsequently asked how often they felt lonely—sometimes or always. The background and demographic variables included respondent's age (*60–69, 70–79, *and* 80s+*), sex (*male, female*), ethnicity (*Malay, Chinese, Indian, *and* Indigenous*), marital status (*currently married, widowed-divorced-separated*), and place of residence (*urban, rural*). The socioeconomic variables for this study included educational level (*no schooling, primary, *and* secondary and above*), work status (*still working, stopped working*), and number of sources of income (*none, 1-2 sources, *and* 3+ sources*). The sources of income included (1) inheritance (house, company, land, etc.), (2) savings in the Employees Provident Fund, (3) pension, (4) rewards/remunerations, (5) savings in bank, (6) savings in “*tabung haji*” (Malaysian hajj pilgrims fund board), (7) share investments, and (8) insurance.

Respondents were asked if they were suffering from any of the following illnesses: (1) high blood pressure, (2) diabetes, (3) heart disease, (4) arthritis, and (5) asthma. They were also asked if they had physical limitations in carrying out the following daily activities: (1) self-feeding, (2) bathing, (3) getting dressed, (4) going to toilet, (5) exercising, (6) daily housework, (7) attend religious gatherings, and (8) grocery shopping. The number of health and physical limitations was recoded into categorical variables: number of illnesses (*none, 1 illness, *and* 2+ illnesses*) and number of physical limitations (*none, 1-2 limitations, *and* 3+ limitations*).

Besides coresidence with adult children, family support included four common types of support from adult children on a monthly basis: financial support, help with paying of bills, provision of food/other necessities, and help with housework. These different forms of family support were recoded into dichotomous variables (*Yes, No*). Community participation in religious and leisure activities (sports/neighborhood watch/Nongovernmental organizations' (NGO) activities) was also recoded into dichotomous variables.

### 2.3. Analysis

We began by describing the sociodemographic and socioeconomic characteristics, health and disability and coresidence status of the total sample, and separately for males and females. Cross tabulations were run to examine the bivariate associations between the level of loneliness with sociodemographic variables, family support, and community participation. This was followed by ordinal logistic regression using the proportional odds model to assess the relationship between levels of loneliness and factors of sociodemographic and SES, health and physical condition, family support, and community participation [44]. The ordinal logistic regression was repeated for older Malays, Chinese, and Indigenous persons. The analysis was not conducted for older Indians due to the small number of respondents (*n* = 77). Data were analyzed using SPSS for Windows version 19 and weighted according to the ethnic distribution of the population based on the 2010 population census [30].

## 3. Results

Table 1 shows the distribution of respondents by sociodemographic characteristics and other study variables. The respondents were aged between 60 and 97 years, with a mean of 68 years and standard deviation of 6 years. Almost two-thirds (64%) were in their 60s. There were more females than males in the sample (1081 versus 710). The respondents consisted of 45% Malays, 25% Chinese, 4% Indians, and 26% Indigenous people. Majority of respondents were currently married and slightly more respondents were from rural areas. Slightly more than half had no formal schooling, whereas around a tenth studied above the primary level. A quarter of them were still working and three-quarters had at least 1 source of income. 27% of the respondents were not suffering from any of the 5 diseases, and around two-thirds did not have any physical limitations in performing daily activities. About 63% were coresiding with adult children.

There were rather significant differences between the male and female respondents in terms of marital, SES, and health status. Close to four-fifths of male respondents were currently married, as compared to around two-fifths of females. Around two-thirds of male respondents had attended formal schooling compared to around one-third of females. The proportion who was still working was considerably higher among the males as compared to the females (39% versus 16%). The males were also more likely than the females to have at least one source of income (82% versus 71%). Older females were more likely than older males to have poor health and disability. About three quarters of the females had at least one of the five diseases and 36% had at least one physical limitation, whereas the corresponding figures for the males were 68% and 27%, respectively.

From the total of 1791 respondents, a little less than half (46.6%) reported not feeling lonely, one-third (32.5%) sometimes feeling lonely, and about one fifth (20.9%) always feeling lonely (Table 2). The proportion always feeling lonely was higher among the oldest-old (80s+) as compared to the younger ones, females as compared to the males and Indigenous people groups as compared to other main ethnic groups (Table 3). By contrast, the young-old (60s), males and Chinese had the higher proportion of not feeling lonely. Feeling lonely was also more common among those who were divorced or widowed, living in rural areas, had no formal schooling, stopped working, and had no sources of income. Respondents suffering from illnesses or physical limitations were more inclined to feel lonely. The proportion always feeling lonely was lower for those receiving different forms of support from adult children, living with adult children and participating in religious and leisure activities.

The test of parallel lines shows that the significance of the Chi-square statistics was larger than 0.05 (*χ* ^2^ = 33.01, *P* = 0.104). This suggests that the proportional odds assumption is not violated, and the ordinal logit model is the appropriate statistical technique for this analysis [44]. Results from the logit model show that demographic and socioeconomic factors, health, community participation, and family support for the older persons have significant effects on the feelings of loneliness among older persons (Table 4). The odds of feeling more lonely were 70% higher among the oldest-old as compared to the young-old (OR = 1.70, 95% CI: 1.14–2.54) and 49% lower among married as compared to the divorced/widowed (OR = 0.51, 95% CI: 0.41–0.64). Compared to Malay respondents, the odds of feeling more lonely were 64% lower among Chinese (OR = 0.36, 95% CI: 0.28–0.46) and 48% lower among Indian respondents (OR = 0.52, 95% CI: 0.34–0.77), but there was no significant difference between the Indigenous groups and the Malays.

The odds of feeling more lonely among those with 1-2 and 3 or more sources of income were 29% and 43% lower as compared to those with no source of income (OR = 0.71, 95% CI: 0.56–0.89 and OR = 0.57, 95% CI: 0.43–0.77). Compared to those with no health problems, the odds of feeling more lonely were 47% and 65% higher among those suffering from 1 or 2 and 3 or more chronic illnesses (OR = 1.47, 95% CI: 1.15–1.87; and OR = 1.65, 95% CI: 1.30–2.10). In terms of family support, the odds of feeling more lonely were 21% lower among respondents who received monthly monetary support (OR = 0.79, 95% CI: 0.64–0.98) and 33% lower among respondents who lived with children (OR = 0.67, 95% CI: 0.53–0.84), as compared to those who did not. In terms of community engagement, the odds of feeling more lonely were 23% lower among those who engaged in religious activities (OR = 0.77, 95% CI: 0.62–0.96) as compared to those who did not, but participation in leisure activities had no significant effect on the levels of loneliness.

Results from the logit model for each ethnic group (Table 5) show that age had no significant effect on the levels of loneliness, although it was significant in the overall model. Being married was significantly associated with less loneliness among older Malays (OR = 0.67, 95% CI: 0.49–0.92), Chinese (OR = 0.31, 95% CI: 0.19–0.51), and Indigenous persons (OR = 0.48, 95% CI: 0.32–0.72). Educational level was not significant in the overall model, but it was found to be significant among Malays (OR = 0.70, 95% CI: 0.52–0.94 and OR = 0.51, 95% CI: 0.28–0.93) and Indigenous persons (OR = 0.21, 95% CI: 0.05–0.93). The odds of feeling more lonely were lower among those with formal education. Although sources of income had a significant effect on the levels of loneliness in the overall model, it was a significant factor only among older Malays (OR = 0.64, 95% CI: 0.46–0.90 and OR = 0.56, 95% CI: 0.38–0.85) and Chinese (OR = 0.53, 95% CI: 0.32–0.89 and OR = 0.43, 95% CI: 0.21–0.88). Suffering from chronic illnesses was associated with higher levels of loneliness among older Malays (OR = 1.63, 95% CI: 1.15–2.29 and OR = 1.72, 95% CI: 1.23–2.41), older Chinese (OR = 1.99, 95% CI: 1.16–3.41), and older Indigenous persons (OR = 1.76, 95% CI: 1.07–2.92). Suffering from physical limitations was not significant in the overall model, but it was significant among the Indigenous groups (OR = 1.73, 95% CI: 1.09–2.75). When it came to family support, monetary support was not significant for all ethnic groups, but help with housework had a significant effect on the levels of loneliness among older Chinese. The odds of feeling more lonely were 42% lower among older Chinese who received help in housework (OR = 0.58, 95% CI: 0.34–0.99). Although coresidence with adult children was highly significant in the overall model, it was not as significant among older Malays. The odds of feeling more lonely were 41% lower among the Chinese (OR = 0.59, 95% CI: 0.35–1.00) and 43% lower among Indigenous groups (OR = 0.57, 95% CI: 0.35–0.92) who were living with adult children. Participation in religious activities was only significant among older Malays, where the odds of feeling more lonely were 31% lower (OR = 0.69, 95% CI: 0.50–0.94).

## 4. Discussion

The 2004 MPFS showed that every other older Malaysian experienced loneliness, and one in five was always lonely. The oldest-old were most susceptible to loneliness, because many were widowed and had few surviving friends. The high proportion of older people feeling lonely warrants attention from policy makers, families, community members, and researchers, as loneliness can lead to depression, psychosocial and health problems. More research for a better understanding of factors that contribute to feelings of loneliness is needed to inform policy and programs to safeguard the well-being of older people.

Older women were more likely than older men to feel lonely as the former were much more likely than the latter to be widowed, and the absence of a spouse contributed to feelings of loneliness. It is important to note that an increasing number of older people will be without a spouse as Malaysia is witnessing a trend towards nonmarriage and a rise in divorce [45]. In 2010, among those aged 75 and over, 42,111 out of 201,654 men or 21% were widowed, compared to 144,194 out of 245,410 women or 59% [30]. Because widowhood is significantly associated with loneliness, controlling for marital status would have reduced the gender differential in loneliness. Likewise, controlling for SES and health status would have also reduced this gender differential.

Among the ethnic groups, the Indigenous groups were most likely to report being lonely, followed by the Malays, whereas the Chinese were least likely to do so. Part of the ethnic differentials could be due to place of residence as rural residents were more likely to feel lonely compared to their urban counterparts. Data from the 2010 Population Census show that only 45% of the Indigenous groups and 67% of the Malays lived in urban areas as compared to about 90% of the Chinese and Indians [30]. However, the logit model shows that even after adjusting for place of residence and other sociodemographic variables, the odds of feeling lonely remained significantly higher among the Indigenous groups and the Malays. Hence, differences in culture and outlook in life across the various ethnic groups have a strong bearing in feelings of loneliness.

The ethnic differential in loneliness in Malaysia is akin to the difference in loneliness among elders from Mediterranean and non-Mediterranean countries. Older persons in the Mediterranean (Spain, France, Italy, Greece, and Israel) tend to have larger families and more children in the household compared to their non-Mediterranean counterparts (Sweden, Denmark, the Netherlands, Germany, Belgium, Switzerland, and Austria), yet they had indicated a greater sense of loneliness [20]. Loneliness can be described as the discontentment which results from the inconsistency between ideal and perceived relationships [5]. Van Tilburg and colleagues [46] suggested that feelings of loneliness tend to be more prevalent in communal societies where there are higher expectations for social contact. It is likely that Malay and Indigenous elders, whose social networks are rather similar to that of Mediterranean elders, have higher expectations for social contact or support compared to the Chinese and Indians. The above argument may also apply to differentials in the feelings of loneliness between older people in the urban and rural areas and across educational groups.

Among SES indicators, higher education attainment and more sources of income were associated with less loneliness. However, the effects were not consistent across ethnic groups. Interestingly, the effects of higher education attainment led to less loneliness among older Malays and Indigenous persons, whereas more sources of income were associated with less loneliness among older Malays and Chinese. It may be hypothesized that work engagement would contribute to reduced loneliness of older persons as they would be interacting with coworkers. However, the present study shows that employment status has little effect on the state of loneliness. An inspection of the data shows that half of the older persons who were still working were engaged in traditional sectors such as agriculture and fisheries. A cross tabulation (not shown here) shows significant association between sector of employment and levels of loneliness (*χ* ^2^ (2, *n* = 424) = 28.134, *P* = 0.000). Older persons working in traditional sectors were more likely to feel lonely (sometimes lonely: 33.2%, always lonely: 23.2%) as compared to those working in formal sectors (sometimes lonely: 22.5%, always lonely: 9.4%). This implies that working past the age of 60 may not necessarily result in reduced loneliness. With the fundamental shift of the economy from the informal to the formal sector and with the increase in retirement age to 60 years effective 2013, more and more older people will be engaged in the formal sector. Thus, this will contribute to reduced loneliness among those who continue to work, as they will be interacting with others in the work place.

Data from the 2004 MPFS showed that about 63% of older Malaysians lived with their adult children, and this did not change much from the 67% reported in the 1988 Malaysian Family Life Survey II [38]. Coresidence with adult children may be due to economic factors such as high housing cost, and it is also encouraged by the Malaysian government through the institution of social policies such as tax incentives. Coresidence with adult children provides companionship for older people and provides a platform for the continuation of support from adult children. This type of assistance would enable interaction and contact with children, which would indirectly lead to emotional support resulting in less loneliness [21–23]. Coresidence with adult children has also been found to have positive effects on the life satisfaction of older Malaysians [47]. The benefits of this living arrangement on alleviating loneliness among older persons are evidently seen among Chinese and Indigenous groups. Conversely, monetary support from children can positively improve an older person's SES. This support may lead to reduced loneliness, as past research has shown that low SES is a risk factor for loneliness [15]. Interestingly, a sizable proportion of older people living together with their children and receiving monetary support reported being lonely. This indicates that some older people felt neglected by coresiding children who were busy with work, fetching their own children to schools or other social engagements. It could also mean that adult children had not been able to meet the needs of companionship of their parents, at times due to mismatch of intergenerational expectations.

Family support in the form of monetary transfers from adult children to parents is commonly practiced in Malaysia, often as a form of repayment for parental investments in the former's education [48]. Heavy reliance on children for financial resources is also due to limited availability of pensions and public social security schemes in Malaysia. An earlier study has shown that majority of older persons in rural areas are not working and had no pension [37]. The 2004 MPFS showed that a small proportion (12%) of older persons had pensions, and around 22% had savings in the Employees Provident Fund (EPF) [41–43]. The Civil Service Pension Scheme only covers workers in public sectors, whereas the EPF is a mandatory defined-contribution plan for working Malaysians and their employers. As of 2009, the EPF covers about half of labour force population, whereas the Civil Service Pension Scheme covers around 11% [49].

The community and NGOs can also play a role in providing care and support for older people by having intergenerational activities that are appropriate for the urban-rural and sociocultural settings. Establishing community centers or day-care centers for older persons to go to while children are at work will also encourage coresidence between older parents and children. Besides lessening the burden of care-giving on the part of children, it can also improve the well-being through companionship and support for the older persons. Universities and colleges may include visitations of older people as part of the community services program for their students. In implementing programs and strategies to improve the well-being of the older people under the National Policy for Older People, it behoves the Ministry of Women, Family and Community Development and other agencies, including NGOs, to implement programs and strategies to strengthen intergenerational relationships among family members and community support for older people.

Our analysis shows that older people who engaged in religious activities were less likely to experience more loneliness. Though, this effect was significantly apparent among older Malays. This finding corroborates with previous research showing that religiosity has a positive association with subjective well-being among Muslims [50, 51]. Leisure activities, however, were not associated with loneliness. A likely reason for this may be due to reverse causation. It is possible that the feelings of loneliness may generate a defensive form of thinking, which can make it harder for one to engage in social activities [52]. Lonely people are more inclined to experience anxiety, pessimism, and fearful of negative evaluation than people who are not lonely. Moreover, older persons at advanced ages with dwindling circle of friends may find it difficult to start new relationships [53].

Our findings corroborate with findings of past studies on the association between chronic diseases and loneliness [18]. Cardiovascular diseases, chronic respiratory diseases, and diabetes are among the top four noncommunicable diseases which have resulted in the highest number of deaths, especially in low- and middle-income countries [54]. People suffering from chronic diseases often have disabilities which restrict their mobility and deter them from forging relationships, which in turn result in loneliness [15, 17]. Unhealthy lifestyle habits such as poor diet, inadequate amount of physical activity, and excessive use of alcohol and tobacco use are known to increase the risk of suffering from chronic diseases [54, 55]. Thus, health intervention programs will not only encourage healthy diet and lifestyles, but also improve the emotional state and well-being of older persons.

This study has several limitations. The ethnic distribution from the 2004 MPFS deviated somewhat from the population. According to the latest national census, the ethnic distribution of Malaysian population aged 60 and above comprised 48% Malays, 36% Chinese, 7% Indians, and 9% Indigenous people [30]. The wide deviation of the ethnic distribution of the sample from the national population was due to the separate sample selection in Sabah and Sarawak to produce sufficient sample sizes for the two East Malaysian states [41, 42]. We have addressed this sampling issue by introducing the appropriate weight to the data. Apart from the ethnic variable, the sample was rather representative of the older population in terms of place of residence, educational level, and marital status [56]. The wide gender disparity in socioeconomic characteristics such as marital status, employment, and educational level corroborates with findings from past studies. These studies show significantly lower literacy rates, labour force participation rates and income among older females and their dependence on financial assistance from adult children, as compared to older males [57, 58].

Another limitation of this study is that findings based on data from a survey conducted ten years ago may not reflect the current situation, as Malaysia has undergone a decade of rapid socioeconomic changes. Moreover, insufficient cases in the Indian sample did not allow for an examination of the levels of loneliness among older Indians. The use of cross-sectional data does not allow a deeper analysis of the changes in the psychosocial behaviour among older Malaysians. However, we stress that the focus of this study is on the effects of family support and social participation on feelings of loneliness within the multiethnic and multicultural context, which has not been studied before. We are of the view that factors affecting loneliness would not have changed substantially in the short run despite the social changes that have taken place. Moreover, this study will provide a useful baseline for a comparative analysis in the future, with the release of the 2014 Malaysian Population and Family Survey data in the near future.

## 5. Conclusion

Our findings reaffirm the important role of the family in alleviating the feelings of loneliness among the elderly. Adult children, especially those who live together, provide physical, financial, and emotional support to their parents. The government has always held that it is the responsibility of the families to take care of their elders. However, family support for the elderly and coresidence may be eroding due to sociodemographic changes such as the trend towards delayed and nonmarriage, shrinking family size, outmigration of the children, increased female labour force participation, and living in condominium [30, 43, 56]. Hence, appropriate programmes and strategies will have to be put in place to strengthen the family institution in the care of the elderly.

## Figures and Tables

**Table 1:** Sociodemographic characteristics, health and disability, and coresidence status of respondents (%).

	All	Male	Female
	(*n* = 1791)	(*n* = 710)	(*n* = 1081)
Age group			
60s	64.4	62.5	65.7
70s	29.2	31.3	27.8
80s+	6.4	6.2	6.5
Gender			
Male	39.6		
Female	60.4		
Ethnic group			
Malay	45.4	41.8	47.8
Chinese	24.6	26.9	23.0
Indian	4.3	3.7	4.7
Indigenous	25.7	27.6	24.4
Marital status			
Currently married	52.8	78.3	36.0
Widowed/divorced	47.2	21.7	64.0
Residence			
Rural	57.0	57.7	56.4
Urban	43.0	42.3	43.6
Educational level			
No schooling	52.4	31.7	66.0
Primary level	36.5	49.4	27.9
Secondary+	11.1	18.9	6.0
Work status			
No	74.7	60.7	83.8
Yes	25.3	39.3	16.2
Sources of income			
None	25.0	18.2	29.4
1-2	52.6	48.5	55.3
3+	22.4	33.4	15.3
Illnesses			
None	27.0	32.1	23.6
1 illness	33.4	32.8	33.9
2+ illnesses	39.6	35.1	42.6
Physical limitations			
None	67.4	73.0	63.7
1-2 limitations	19.5	17.7	20.6
3+ limitations	13.1	9.3	15.6
Coresidence with children			
No	37.2	39.9	35.5
Yes	62.8	60.1	64.5

**Table 2:** Percentage and frequency distribution of respondents by levels of loneliness.

Loneliness	*n*	Percent
Total	***1791***	***100.0***
Not lonely	835	46.6
Sometimes lonely	582	32.5
Always lonely	374	20.9

**Table 3:** Percentage distribution of respondents by levels of loneliness, according to selected sociodemographic and socioeconomic characteristics, health and physical condition, and various forms of family support and community engagement (*n* = 1791).

Variables/categories	Not lonely	Sometimes lonely	Always lonely	Total	*n*
Age group∗∗					
60s	49.5	31.5	19.1	100	1154
70s	44.2	33.5	22.4	100	523
80s+	28.9	38.6	32.5	100	114
Sex∗∗					
Male	54.6	28.9	16.5	100	710
Female	41.4	34.9	23.8	100	1081
Ethnic group∗∗					
Malay	41.2	37.3	21.5	100	814
Chinese	68.0	23.4	8.6	100	440
Indian	57.1	24.7	18.2	100	77
Indigenous	34.1	33.9	32.0	100	460
Marital status∗∗					
Currently married	54.7	30.6	14.7	100	945
Widowed/divorced	37.6	34.6	27.8	100	846
Residence∗∗					
Urban	56.8	28.9	14.3	100	771
Rural	38.9	35.2	25.9	100	1020
Education level∗∗					
No schooling	38.1	34.1	27.8	100	939
Primary level	52.5	33.4	14.1	100	653
Secondary+	67.3	22.1	10.6	100	199
Work status					
Still working	49.3	30.6	20.0	100	454
Not working	45.7	33.1	21.2	100	1337
Sources of income∗∗					
None	37.6	35.6	26.8	100	447
1-2 sources	47.1	32.2	20.7	100	942
3+ sources	55.5	29.9	14.7	100	402
Illnesses∗∗					
None	55.3	29.6	15.1	100	483
1 illness	44.4	31.7	23.9	100	599
2+ illnesses	42.6	35.1	22.3	100	709
Physical limitations∗∗					
None	51.4	30.4	18.1	100	1207
1-2 limitations	37.5	36.7	25.8	100	349
3+ limitations	35.3	37.0	27.7	100	235
Monetary support∗∗					
Yes	50.5	32.0	17.4	100	986
No	41.9	33.0	25.1	100	805
Paying bills∗					
Yes	48.6	33.9	17.5	100	954
No	44.3	30.9	24.7	100	837
Food/other necessities					
Yes	47.2	33.2	19.5	100	1188
No	45.4	31.0	23.5	100	603
Housework∗					
Yes	46.6	34.8	18.6	100	1102
No	46.7	28.7	24.5	100	689
Coresidence∗∗					
Yes	50.3	33.1	16.6	100	1124
No	40.5	31.6	27.9	100	659
Religion activities					
Yes	46.6	34.0	19.4	100	977
No	46.5	30.9	22.6	100	810
Leisure activities					
Yes	46.5	33.1	20.4	100	824
No	46.7	32.0	21.3	100	967

Note: ∗∗*P* < 0.001; ∗*P* < 0.05.

**Table 4:** Results of ordinal logistic regression on higher levels of loneliness (*n* = 1791).

Attributes	OR	95% CI
Age group (*ref: 60s*)		
70s	0.99	(0.79–1.24)
80s+	**1.70**	**(1.14–2.54)**
Male	0.95	(0.75–1.20)
Ethnicity (*ref: Malay*)		
Indigenous	1.25	(0.90–1.73)
Chinese	**0.36**	**(0.28–0.46)**
Indian	**0.52**	**(0.34–0.77)**
Married	**0.51**	**(0.41–0.64)**
Urban	0.81	(0.65–1.00)
Edu level (*ref: no schooling*)		
Primary level	0.83	(0.67–1.04)
Secondary^+^	0.81	(0.57–1.17)
Still working	0.99	(0.78–1.27)
Income sources (*ref: none*)		
1-2 sources	**0.71**	**(0.56–0.89)**
3+ sources	**0.57**	**(0.43–0.77)**
Illnesses (*ref: none*)		
1 illness	**1.47**	**(1.15–1.87)**
2+ illnesses	**1.65**	**(1.30–2.10)**
Physical limitations (*ref: none*)		
1-2 limitations	1.16	(0.90–1.49)
3+ limitations	1.13	(0.84–1.52)
Monetary	**0.79**	**(0.64–0.98)**
Payment of bills	0.99	(0.78–1.25)
Food/necessities	0.85	(0.66–1.11)
Housework	0.95	(0.74–1.21)
Coresidence	**0.67**	**(0.53–0.84)**
Religious activities	**0.77**	**(0.62–0.96)**
Leisure activities	1.00	(0.81–1.24)
Chi-square^a^	33.010	
*P* value^a^	0.104	
*df* ^ a^	24	

Note: odds ratio in bold indicates significance at 0.05.

^a^Test of parallel lines.

**Table 5:** Results of ordinal logistic regression for ethnic groups.

Attributes	OR (95% CI)		
	Malay	Chinese	Indigenous
	(*n* = 814)	(*n* = 440)	(*n* = 460)
Age group (*ref: 60s*)			
70s	0.84 (0.60–1.17)	1.10 (0.66–1.84)	1.01 (0.66–1.56)
80s+	1.69 (0.92–3.09)	1.53 (0.67–3.52)	1.31 (0.62–2.78)
Male	0.78 (0.55–1.11)	1.25 (0.74–2.11)	0.76 (0.50–1.18)
Married	**0.67 (0.49–0.92)**	**0.31 (0.19–0.51)**	**0.48 (0.32–0.72)**
Urban	0.83 (0.62–1.10)	0.81 (0.47–1.39)	0.78 (0.47–1.29)
Edu level (*ref: no schooling*)			
Primary level	**0.70 (0.52–0.94)**	1.02 (0.61–1.72)	0.86 (0.48–1.53)
Secondary^+^	**0.51 (0.28–0.93)**	1.17 (0.58–2.34)	**0.21 (0.05–0.93)**
Still working	1.14 (0.80–1.63)	0.64 (0.35–1.14)	1.01 (0.66–1.55)
Income sources (*ref: none*)			
1-2 sources	**0.64 (0.46–0.90)**	**0.53 (0.32–0.89)**	0.99 (0.66–1.47)
3+ sources	**0.56 (0.38–0.85)**	**0.43 (0.21–0.88)**	0.91 (0.49–1.71)
Illnesses (*ref: none*)			
1 illness	**1.63 (1.15–2.29)**	1.12 (0.65–1.94)	**1.76 (1.07–2.92)**
2+ illnesses	**1.72 (1.23–2.41)**	**1.99 (1.16–3.41)**	1.05 (0.63–1.73)
Physical limit (*ref: none*)			
1-2 limitations	1.23 (0.87–1.74)	1.25 (0.65–2.38)	**1.73 (1.09–2.75)**
3+ limitations	1.26 (0.84–1.89)	1.39 (0.66–2.93)	1.31 (0.68–2.53)
Monetary	0.78 (0.58–1.06)	0.79 (0.48–1.30)	0.98 (0.62–1.53)
Payment of bills	0.87 (0.61–1.23)	1.16 (0.66–2.05)	0.65 (0.41–1.02)
Food/necessities	0.98 (0.67–1.44)	0.72 (0.40–1.30)	0.77 (0.44–1.34)
Housework	1.22 (0.85–1.76)	**0.58 (0.34–0.99)**	0.96 (0.56–1.62)
Coresidence	0.73 (0.53–1.01)	**0.59 (0.35–1.00)** ^ +^	**0.57 (0.35–0.92)**
Religious activities	**0.69 (0.50–0.94)**	0.92 (0.57–1.49)	1.05 (0.71–1.55)
Leisure activities	1.08 (0.81–1.45)	1.06 (0.64–1.77)	1.02 (0.69–1.51)
Chi-square^a^	32.244	9.461	20.948
*P* value^a^	0.550	0.985	0.462
*df* ^ a^	21	21	21

Note: odds ratio in bold indicates significance at 0.05.

^a^Test of parallel lines.

^+^Marginal significance.
